# The effects of natural structure on estimated tropical cyclone surge extremes

**DOI:** 10.1007/s11069-017-2935-y

**Published:** 2017-05-31

**Authors:** Donald T. Resio, Taylor G. Asher, Jennifer L. Irish

**Affiliations:** 1 University of North Florida, Jacksonville, FL USA; 2 University of North Carolina, Chapel Hill, NC USA; 3 Virginia Tech, Blacksburg, VA USA

**Keywords:** Storm surges, Probabilities, Tropical cyclone hazards, Surge extremes

## Abstract

The past 12 years have seen significant steps forward in the science and practice of coastal flood analysis. This paper aims to recount and critically assess these advances, while helping identify next steps for the field. This paper then focuses on a key problem, connecting the probabilistic characterization of flood hazards to their physical mechanisms. Our investigation into the effects of natural structure on the probabilities of storm surges shows that several different types of spatial-, temporal-, and process-related organizations affect key assumptions made in many of the methods used to estimate these probabilities. Following a brief introduction to general historical methods, we analyze the two joint probability methods used in most tropical cyclone hazard and risk studies today: the surface response function and Bayesian quadrature. A major difference between these two methods is that the response function creates continuous surfaces, which can be interpolated or extrapolated on a fine scale if necessary, and the Bayesian quadrature optimizes a set of probability masses, which cannot be directly interpolated or extrapolated. Several examples are given here showing significant impacts related to natural structure that should not be neglected in hazard and risk assessment for tropical cyclones including: (1) differences between omnidirectional sampling and directional-dependent sampling of storms in near coastal areas; (2) the impact of surge probability discontinuities on the treatment of epistemic uncertainty; (3) the ability to reduce aleatory uncertainty when sampling over larger spatial domains; and (4) the need to quantify trade-offs between aleatory and epistemic uncertainties in long-term stochastic sampling.

## Introduction

The estimation of storm surges, from both tropical and extratropical cyclones, is critical to quantifying hazards, risks and resilience in coastal areas. In the aftermath of Hurricane Katrina, extensive efforts were funded and major progress was made in this area (Westerink et al. 2008; Resio et al. 2008; Irish et al. 2009; Niedoroda et al. 2010; Toro et al. 2010; Irish and Resio 2010). These new methods represented significant advances over the previous approaches which were based only on hindcasts of historical or hypothetical “design storms” to estimate storm surges in an area. However, a situation that often emerges following such advances is that substantial time can pass before additional research needs are recognized. During such periods, approximations and procedures initially justified to meet necessary operational time and computational limitations can become adopted as final solutions for complex problems. Knowledge gained about potential deficiencies in these methods, if not documented, can become lost through time before the next cycle of major studies is undertaken. Consequently, one goal of this paper is to provide a review to help minimize this potential knowledge gap.

It is well known that nature is organized within a hierarchy of spatial and temporal scales that govern atmospheric and oceanic motions (Kraus and Businger 1994; Wells 2011; Vallis 2014). Within this natural structure, smaller-scale circulation systems are nested within larger-scale systems that play an important role in the motions and intensities of the smaller scales. As an example of this organization, many larger-scale atmospheric circulations patterns (e.g., North Atlantic Oscillation, El Niño, the influence of land–sea boundaries) influence patterns of motions and intensities of synoptic-scale storms. Additionally, coastal configuration, bathymetry and topography create systematic variations of flooding characteristics within individual coastal areas via different responses to a given set of forcing processes. In this paper, potential effects of natural structure on surge frequencies and magnitudes from tropical cyclones are investigated, with a focus on effects outside of those typically treated in statistical approaches used today. For example, typical estimates of inundation hazards assume that storm characteristics in a given region are drawn from a single stationary, homogeneous population. In this context, known physical constraints on the energy balance in obliquely landfalling storms and the effects of extended episodes of low storm activity and high storm activity are often neglected. This paper investigates potential effects of such natural structure in estimates of statistical storm surge hazards used today for setting coastal insurance rates and for community planning decisions. In Sect. 2, we present a brief summary of historical methods and their evolution to the present state of the art. This is then used for perspective in discussing the roles of and bases for today’s methods being applied in many studies. Sections 3 and 4 examine specific effects of natural structure on hazard estimates, with Sect. 3 addressing the effects of assumptions which neglect physical factors and Sect. 4 addressing effects of natural structure that are related to the treatment of uncertainty in hazard/risk estimation. In Sect. 4, as commonly accepted in hazard and risk analysis today (National Academy 1997), we separate the uncertainty analyses into two parts: epistemic and aleatory. Although the definitions of epistemic and aleatory uncertainty are relatively straightforward, their interpretations are often somewhat contentious, a point not lost in the review by the National Academy which points out that distinctions used in such classification tend to be quite ambiguous.

As defined in by Der Kiureghian and Ditlevsen (2009):

Uncertainties are characterized as epistemic, if the modeler sees a possibility to reduce them by collecting more data or by refining models. Uncertainties are categorized as aleatory if the modeler does not foresee the possibility of reducing them. From a pragmatic standpoint, it is useful to categorize the uncertainties within a model, since it then becomes clear to us which uncertainties have the potential of being reduced.

As noted subsequently in their paper, they emphasize the point that the need for distinction between these two terms is more relevant to “risk communication” than it is to “risk assessment.” As long as all significant contributors to uncertainty are considered, the values obtained for risk assessment should remain the same, regardless of how they are categorized. For the purpose of this paper, we include uncertainties that involve approximations to model input that are model dependent, such as wind-field parameterizations and surge model accuracies, as epistemic. We treat estimates of characteristics that depend on the number of samples, such as number of historical storms in an area, best-fit statistical parameters and the period of record, as aleatory.

## Methodologies used to quantify coastal surge hazards

In the 1960s, due to the paucity of reliable data and lack of computational resources, most coastal hazards were still being quantified via design storms, for example the Standard Project Hurricane (SPH) and the Probable Maximum Hurricane (PMH) (Graham and Nunn 1959; Schwerdt et al. 1979). By the 1970s, computer capabilities and models for surges and waves proliferated (Welander 1961; Reid and Bodine 1968; Jelesnianski 1967; Leendertse 1967; Bunting 1970). This initiated a transition from design storm scenarios to hindcasts of sets of historical storms combined with an analysis of computed extreme values. By the late 1980s, hindcasts of both surges and waves generated by storms were commonplace, and the focus of optimal methods for quantifying hazards shifted to various statistical methods used for analyzing extremes. Although usage of design storms has persisted in some applications, more robust statistical frameworks have become the norm.

Four fundamentally different approaches to the statistical problem of tropical cyclone storm surge extremes have now been hypothesized and applied in various studies. In this section, we present historical background on the methods and then discuss some key differences between them. As will be shown, many of these methods are not mutually exclusive, but instead solve different parts of the same problem.

1. The historical storm method (HSM) uses parametric fits to historical surge data, usually obtained from water-level gages or numerical models forced by reconstructions of historical wind fields (Reid et al. 1975; Crawford 1979).
2. The joint probability method (JPM) combines a set of parameters to characterize tropical cyclone wind and pressure fields with a multivariate distribution (of those parameters) to represent a set of all storms that could occur in an area (e.g., Ho et al. 1975; Myers 1975). The JPM with optimal sampling (JPM-OS) introduces a modification to the JPM where an optimized subset of the combinations within the continuous distribution is used to define the surge response at a given site (e.g., Resio et al. 2008; Irish et al. 2009; Toro et al. 2010).
3. The empirical simulation technique (EST) is basically the HSM as described above, combined with nonparametric resampling to provide estimates of variability within the range of return periods covered by the samples but still requires extrapolation to longer return periods (Scheffner et al. 1996).
4. The stochastic-deterministic track method (SDTM), an extension of the initial stochastic track method (STM) of Vickery and Twisdale (1995), uses a combination of physically based models and stochastic tropical storm track behavior to create a very large set of synthetic storms (Emanuel et al. 2006).

### The historical storm method (HSM)

Analogous to methods used in hydrological analyses for historical datasets (Langbein 1949; Beard 1962; Chow 1964; US Water Resources Council 1967; Beard 1974), initial HSMs used peak water levels from annual records or hindcasts combined with parametric fitting techniques, typically based on rank–order plotting position, to estimate annual exceedance probability (AEP) or return period (*T*_R_),

1 $${\text{AEP}}\left( x \right) = 1 - F\left( x \right),\quad {\text{or}}\quad T_{\text{R}} \left( x \right) = \frac{1}{{{\text{AEP}}\left( x \right)}} = \frac{1}{1 - F\left( x \right)}$$

where *x* is the surge height at a fixed point and *F*(*x*) is the cumulative distribution function (CDF). Note that both *T*_R_ and the AEP should be viewed in terms of the frequency of exceedance, rather than a fixed interval between exceedances, since the interval between exceedances is a random quantity. Two approaches are typically used in selecting extreme events: the block maxima (e.g., Gumbel 1934; Jenkinson 1955) approach or the peaks-over-threshold approach (Pickands 1975; Davison and Smith 1990; Coles 2001). An extension of the block maxima approach allows for stratification of samples by storm type, using a combined Poisson-generalized extreme value (Poisson-GEV) distribution,

2 $${\text{AEP}}\left( x \right) = \lambda \left( {1 - F\left( x \right)} \right),\quad {\text{or}}\quad T_{\text{R}} \left( x \right) = \frac{1}{{{\text{AEP}}\left( x \right)}} = \frac{1}{{\lambda \left( {1 - F\left( x \right)} \right)}}$$

where *λ* is the Poisson parameter for the average annual frequency of a type of storm, such as a tropical or extratropical cyclone. Note again that this definition combines frequency and probability, since *λ* introduces a time unit here. Since $F(x)$ is dimensionless, the time factor in $\lambda$ must be in years for the ${\text{AEP}}(x)$ to have units of probability of exceedance per year and return period to be in terms of years. The need for $\lambda$ is often not recognized since it is assumed in many fields that the data represent annual maxima and that the units are years.

The peaks-over-threshold approach uses a threshold value and convergence of the best-fit parameters as a criterion to approach the same problem as the Poisson-GEV example above. However, both methods depend on interpolation and extrapolation of parameterized probability distributions using best-fit parameters to estimate values at the desired AEPs and associated return periods, and since many estimates lie beyond the historical record length, they still rely heavily on assumptions of sample independence and the existence of a homogeneous population over the range used in the estimation.

The HSM is purely dependent on historical data, making it highly sensitive to variability therein. As shown by Monte Carlo simulations in Irish et al. (2011), the HSM, based on surge hindcasts of historical storms, produces far more variability in the estimates than the JPM. A discussion of the source of this gain in information will be given in a subsequent section of this paper. Agbley and Basco (2008) and Resio et al. (2008) have both shown that EST’s parametric extrapolation can lead to unstable statistics depending on the available data at a given site.

### The joint probability method (JPM)

By the 1970s, sufficient computer capacity existed to allow simulations of relatively large sets of tropical cyclones leading to the development of early JPM studies (Myers 1975; Ho et al. 1987). These studies characterized tropical cyclone wind fields by a small number of storm parameters and developed relatively ad hoc sampling methods to select a set of combinations of storms considered possible; surge was then simulated for each parameter combination. Existing computer capabilities limited the spatial resolution possible in these studies, and the storm sample available at that time did not allow significant parameter correlations to be incorporated into the probability estimates in most areas, even though this was not an actual constraint within the JPM framework. Another limitation in these early studies was the neglect of uncertainty in hazard/risk assessments. The JPM was used extensively in early FEMA coastal studies in the 1970s and 1980s.

### The empirical simulation technique (EST)

In the 1990s, the nonparametric EST approach was developed for estimating *F*(*x*) (Scheffner et al. 1996), using resampling methods. Although this method was nonparametric within about half the period of record and provided an excellent means of investigating sampling variability via resampling, EST still relied on parametric extrapolations to extend the CDF beyond the record length. Following Hurricane Katrina in 2005, it became apparent that it was important to consider surge from all storms that could occur in an area and not just those that had occurred within that area’s historical record. The apparent insufficiency in the historical record shifted the primary approach to coastal surge hazards away from the EST and back toward the JPM.

### The stochastic-deterministic track method (SDTM)

Vickery and Twisdale (1995) introduced the STM, an alternative methodology for applications to hurricane wind hazards within the USA. Unlike the JPM, which uses an ordered set of tracks such that a sequence of each parameter is combined with prescribed values of all the other parameters, the STM uses random seeding of initial tropical cyclone genesis followed by stochastic predictions of track and other parameters developed from empirical relationships derived from historical datasets. This STM is used to simulate a randomly generated set of storms for each year of simulated time. Emanuel et al. (2006) introduced the SDTM by extending the STM to include a stronger physical basis for hurricane development and decay, including a dependence on large-scale circulation conditions within an idealized model of hurricane energy sources and sinks. The primary objective of this approach is to allow more physics to be encapsulated within the statistics and to be able to extend the sampling interval using resampling methods. This has the effect of decreasing the dependence on the historical record and allowing for hypothetical scenarios, such as climate change, to be explored.

In both the STM and SDTM, many synthetic storms are generated over a long simulation interval. For simulations in which the computational requirements are minimal (such as parametric wind-field estimations), this does not create any real problem. Unfortunately, high-resolution coupled surge–wave hydrodynamic models (hereafter “surge models” for brevity) used to simulate coastal storm surges require very extensive computer resources (hundreds to thousands of computational hours per storm simulation), which represents a major obstacle to the utilization of this approach in surge hazard estimation. So far, use of the STDM has been limited to academic studies (e.g., Lin et al. 2010). It should be noted, however, that an important advantage of Emanuel’s SDTM approach is that it does allow the incorporation of potential climate change directly into the hazard definition and that information from the SDTM tracks could potentially be distilled into a more manageable set for simulations.

### The joint probability method with optimal sampling (JPM-OS)

Following the 2004 and 2005 Atlantic hurricane seasons, it was evident that improved physics, numerical methods and model resolution were all critical to accurate inundation estimates, particularly in inland areas and where river discharges couple significantly to inland flooding (Resio and Westerink 2008; Westerink et al. 2008; Dietrich et al. 2011, etc.). Unlike forecast methods, where very large track and intensity uncertainties limit accuracy and result in the need for relatively high levels of conservatism for safety considerations, hindcast surge simulations must be as accurate as possible to enable reasonable, unbiased assessments of hazards and risks for design, planning and actuarial uses. These stringent modeling requirements make it important to rigorously treat both the statistics and the surge modeling.

Resio et al. (2008) introduced a modification to the JPM to simultaneously leverage the method’s statistical stability and the advent of accurate, high-fidelity surge modeling capabilities. This is achieved via what has been termed “optimal sampling” (JPM-OS), where a limited number of high-fidelity surge simulations are used to specify surges in the multivariate JPM integral. Since the JPM-OS’s introduction, the selection of the optimal storm set has been relatively ad hoc. Thus, it is important to revisit the initial conceptual basis for choosing optimal sets of storms for high-resolution surge modeling. Two methods have been utilized post-Katrina: the JPM-OS-surge response function (JPM-OS-SRF) and the JPM-OS-Bayesian quadrature (JPM-OS-BQ). Both methods have advantages and disadvantages, as will be discussed subsequently in this paper.

High-resolution, coupled wave–surge models used for accurate calculation of surges in coastal areas require large computer run times even for the simulation of a single storm. The development of methods to minimize computational resources needed for calculations of surge probabilities was critical to the ability of the JPM approach using these models to reduce the number of simulations to an acceptable limit for practical applications. The SRF uses continuous spatial functions for its basis to minimize the number of storms need to be simulated, while the BQ uses a finite set of points within the storm parameter space for this purpose. It is probably more accurate to refer to these two approaches as JPM-optimized sampling methods, rather than the JPM-optimal sample methods. Since additional storms will asymptotically increase the accuracy of the statistical results, the selected set in either the SRF approach or BQ approach does not represent the minimum achievable residual, but only estimates of a set that meets some accuracy thresholds within an achievable number of simulations.

## Some basic differences in methodologies

It is important to recognize basic distinctions among the methods described above, since these can affect their interpretation and suitability for specific applications. Each method attempts to solve the probabilistic flood hazard problem in a somewhat different fashion and employs different tools to develop storm sets and will be discussed in this section.

### Probability mass versus continuous distributions

Probabilities using the HSM are typically defined in terms of probability masses. In univariate probability distributions, this is accomplished either by using plotting positions for the CDF or by using alternative methods (maximum likelihood, maximum entropy, resampling, L-moments, etc.) to define a continuous CDF,

$$\int\limits_{0}^{x} {p(\hat{x}){\text{d}}\hat{x}} = F(x) \to \sum\limits_{i = 1}^{n} {p(\hat{x}|\hat{x} < x)\delta \hat{x}} .$$

For a case with *n* events in the summation, a set of sample values can be directly interpreted in terms of probability masses, after ranking the samples and choosing an estimate of *F*(*x*) in terms of its position within the sample. For example, $F(x) = (n) /(N + 1), \, (n - 0.5) /N$ or other distribution-dependent plotting positions can be derived and utilized. A particular difficulty with the HSM is the assumption that the storms are all drawn from a stationary, homogeneous parent population. The incorporation of mixed populations within a single sample has long been recognized as a major potential pitfall in the use of historical storms (Resio et al. 2017). Even in situations where a clear distinction often appears to exist, such as extratropical storms versus tropical cyclones, this separation can be completely artificial in its inclusion of hybrid storms within either category. A more subtle impact related to spatial structure emerges when surges from the periphery of storms are combined with surges from the intense cores of the storms, since the statistical behavior of storm intensity is not generally related to the same statistical distribution as the spatial gradients along these storms. Furthermore, subdivision of the already sparse historical record can exacerbate problems in parametric data fitting.

Some applications have assumed that a historical set of storm observations can be directly extended to samples within a multivariate context, since we can write

$$\int\limits_{0}^{x} {p(\hat{x}_{1} ,\hat{x}_{2} , \ldots ,\hat{x}_{n} ){\text{d}}\hat{x}_{1} ,{\text{d}}\hat{x}_{2} , \ldots ,{\text{d}}\hat{x}_{n} } = F(x) \to \sum\limits_{i = 1}^{n} {p(x_{1} ,\hat{x}_{2} , \ldots ,\hat{x}_{n} |\Phi (\hat{x}_{1} ,x_{2} , \ldots ,x_{n} ) < x)\delta \hat{x}_{1} ,\delta \hat{x}_{2} , \ldots ,\delta \hat{x}_{n} } .$$

where $\Phi (\hat{x}_{1} ,\hat{x}_{2} , \ldots \hat{x}_{n} )$ is a numerical operator that converts a set of parameter values into a scalar variable (surge elevation) which in most cases today is represented by a numerical surge model driven by a parametric wind field. In HSM and SDTM applications, the “*n*” storms included in a sample are directly related to probabilities by their relative frequency over the time observed/simulated. The representation of these probabilities and their surges as a finite set of discrete realizations allows the maximum storm surge at each modeled point to be ranked and associated with a specific return period. However, since parameters used to define the multivariate probability space are continuous, surges generated are more accurately represented, at least in a physical context, as points within the continuous multivariate distributions. When treated as a set of discrete events, the probability mass assigned to an event depends not only on the specific parameters used to categorize the event in a multivariate probability space, but also on the size of the probability space assigned to that event.

The problem with limited sampling intervals for historical storms has been approached via extending the sampling time in the SDTM. This method retains the same concept of probability masses as the HSM but extends the sampling interval using randomized, synthetic storms over a longer interval than the historical record; however, as will be discussed later, the potential reduction in aleatory uncertainty may be offset by increased epistemic uncertainty inherent in both the storm simulation methods and the lower-resolution surge models used in this approach.

The JPM-OS-BQ uses probability masses in its representation of the surge probability integral within the general concept of Gaussian quadrature (GQ)/Bayesian quadrature (BQ) (Yarvin and Roklin 1998; Minka 2000). The BQ version has been used extensively in recent large-scale FEMA studies and is described by Toro (2008), Toro et al. (2010) and Niedoroda et al. (2010). The key difference between the BQ approach and the GQ is the introduction of “influence functions” (linear scaling functions in this case) that are used to guide the sampling pattern in the BQ.

The JPM-OS-SRF uses multivariate probability distributions of tropical cyclones parameters to define a probability density at any point within the simulated multidimensional parameter space. In this approach, the estimated probability mass around a point depends on the arbitrary incremental elements centered on the point. Any single point in this space has zero probability since it has no integration volume, i.e., $p(x_{1} ,x_{2} , \ldots ,x_{n} )\delta x_{1} \delta x_{2} \ldots \delta x_{n} \to 0, \, as \, \delta x_{1} \delta x_{2} \ldots \delta x_{n} \to 0$, which means that there will be zero probability if any of the parameters are not given a finite width. However, such discretization is arbitrary and a single storm should not be interpreted as a probability mass unless it is derived in such a manner that resolves important features within the response surface. These functions have been studied and quantified in several studies as shown in Resio et al. (2008), Irish et al. (2009), Irish and Resio (2010) and Taylor et al. (2015), and in a broad sense, these functions can be considered to be prior information in the same context as the “scale factors” introduced into the BQ method. Since these functions are defined as continuous scalable, locally fit functions, their values can be defined in terms of the continuous parameters of the surge model simulations. Thus, the probability increments used in the JPM-OS-SRF integral are much smaller than discretized increments used in the set of simulated storms. An application of the SRF approach in a probability mass context that treats the set of storms as a fixed set of probability masses and does not interpolate/extrapolate the results to a finer, more extensive basis during the evaluation of the probability integral can lead to bias in estimated surge probabilities (Fishbach et al. 2016); however, such applications of the SRF does not conform to its intended usage.

In a general sense, all physical information available to define tropical cyclone surges at given locations in the JPM is derived from physics-based surge models, while the statistical information is inherent to the probabilities of combinations of hurricane parameters associated with surges at a given spatial location. The BQ method, neglecting uncertainty, combines these two aspects of the probability integral by finding a single optimal form for an integral of the form

$${\text{AEP}}(\eta ) \approx \sum\limits_{i = 1}^{n} {\lambda_{i} } P[\hat{\eta }_{i} > \eta ]$$

where *λ*_*i*_ is the annual occurrence rate for the *i*th storm in the optimized set and $\hat{\eta }_{i}$ is the modeled surge value at the point of interest by generated by the *i*th storm, with $P[\hat{\eta }_{i} > \eta ]$ being equivalent to a Heaviside function, $H(\hat{\eta }_{i} - \eta )$, i.e., equal to 1 when $\hat{\eta }_{i} > \eta$ and equal to 0 otherwise.

The “Reference Set” used for this purpose is run at substantially lower resolution than the resolution used in simulations for flood mapping, and limiting the optimization is to a relatively small swath of points at or near the coast. Estimated values of the $\lambda_{i}$ values within this approach are optimal in the sense that, for a given number of selected storms, they produce the minimum residuals in the exceedance probabilities obtainable using weighted linear sums of terms.

**Fig. 1:**
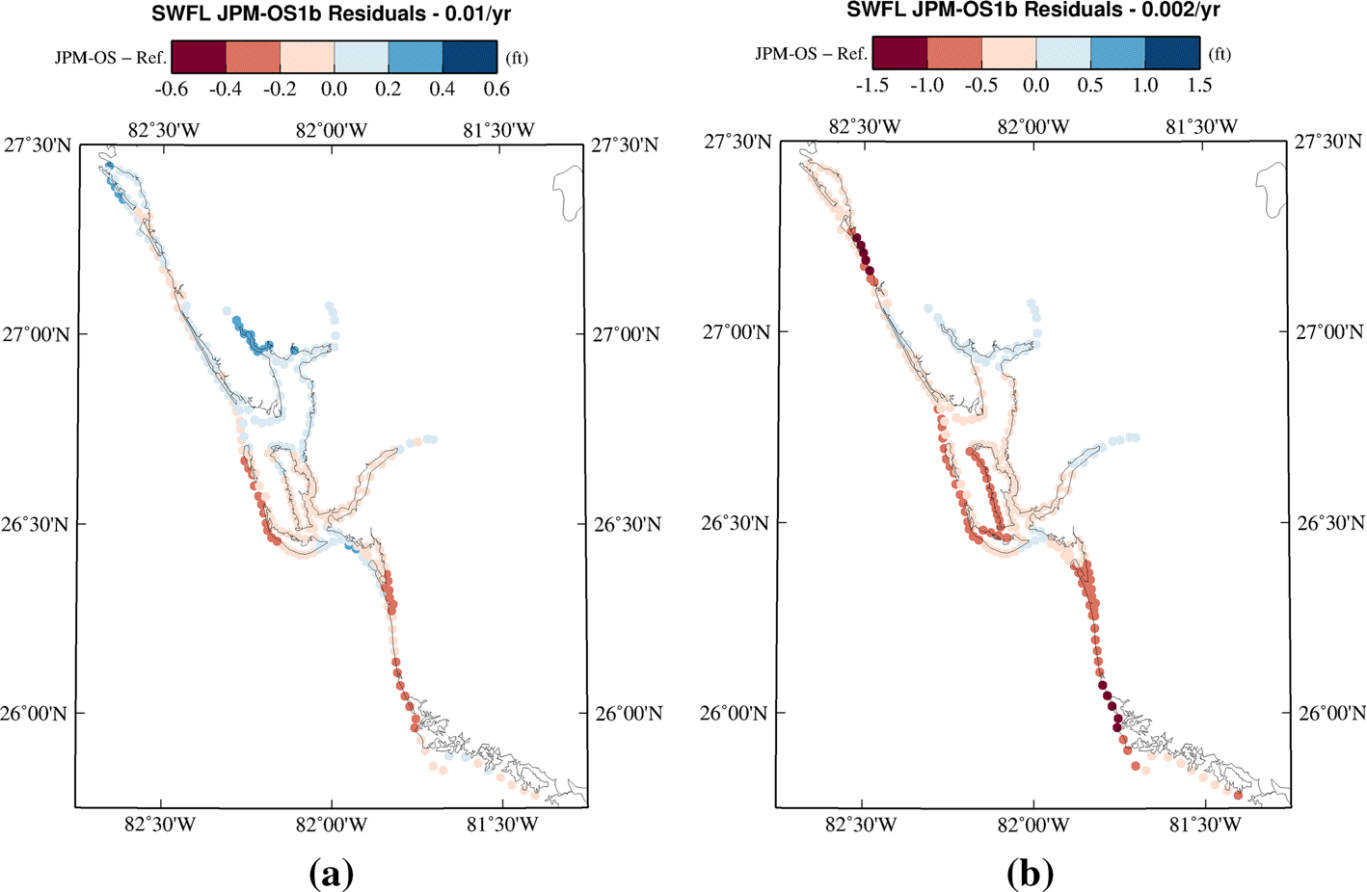
**a** Deviations between estimated surge levels associated with AEP of 0.01 along the coast and in Tampa Bay produced by Bayesian quadrature storm set and “Reference Set” storms. **b** Deviations between estimated surge levels associated with AEP of 0.002 along the coast and in Tampa Bay produced by Bayesian quadrature storm set and “gold-standard” storm set

As with all optimizations dependent on distances between estimators and estimated values, problems can emerge when clusters of points deviate from general behavior or if large-scale trends exist within the data. Figure 1a, b shows results of a comparison of estimates from a BQ application in the Charlotte Harbor area for the AEPs equal to 0.01 and 0.002. This study focused its optimization on the 0.01 AEP, leading to its higher accuracy at that level, yet both suffer from the same large-scale spatial variations in error. These patterns are commonly seen in BQ-based studies that attempt to optimize the storm set homogeneously across the study area, suggesting that moving beyond this shortcoming is a key step in improving the performance of the BQ approach.

**Fig. 2:**
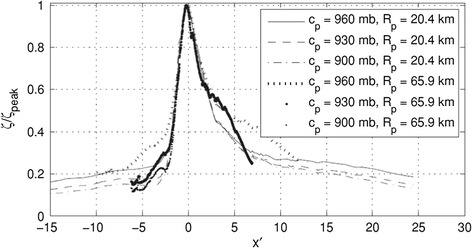
Alongshore variation in dimensionless surges scaled by the peak alongshore value $(\eta /\eta_{\hbox{max} } )$ from Irish et al. (2009). *x′* = (*x* − *x*_*o*_)/*R*_*p*_, where *x* is the station position, *x*_*o*_ is the landfall location, and *R*_*p*_ is the storm radius

**Fig. 3:**
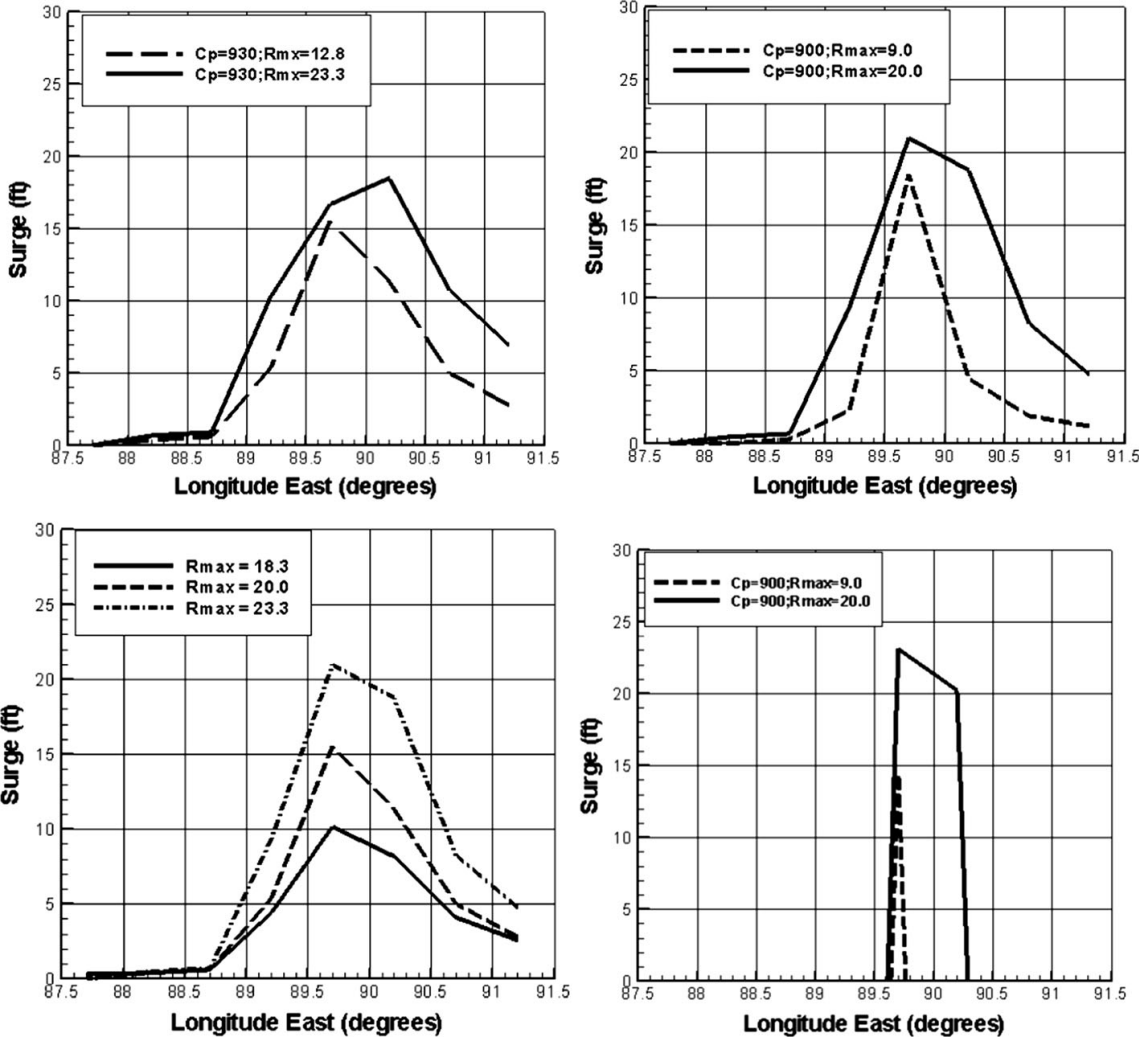
Along-coast patterns of surges from the “gold standard” developed using SLOSH along the Mississippi coast

Figure 2 shows the pattern of surges as a function of track location along the coast from Irish et al. (2009). As can be seen here, the pattern of surges in such areas is very nonlinear with a well-defined region of maximum value and a variation of about 10% within a distance of ±0.5 rmax from the peak value. Figure 3 shows examples of surges from simulations used to create the Reference Set of storms along the Mississippi coast using the SLOSH model. Even at the fine-scale spacing of storm tracks used in this Reference Set from (Niedoroda et al. 2010), it is clear that the along-coast surge maxima can be truncated by spatial discretization.

**Fig. 4:**
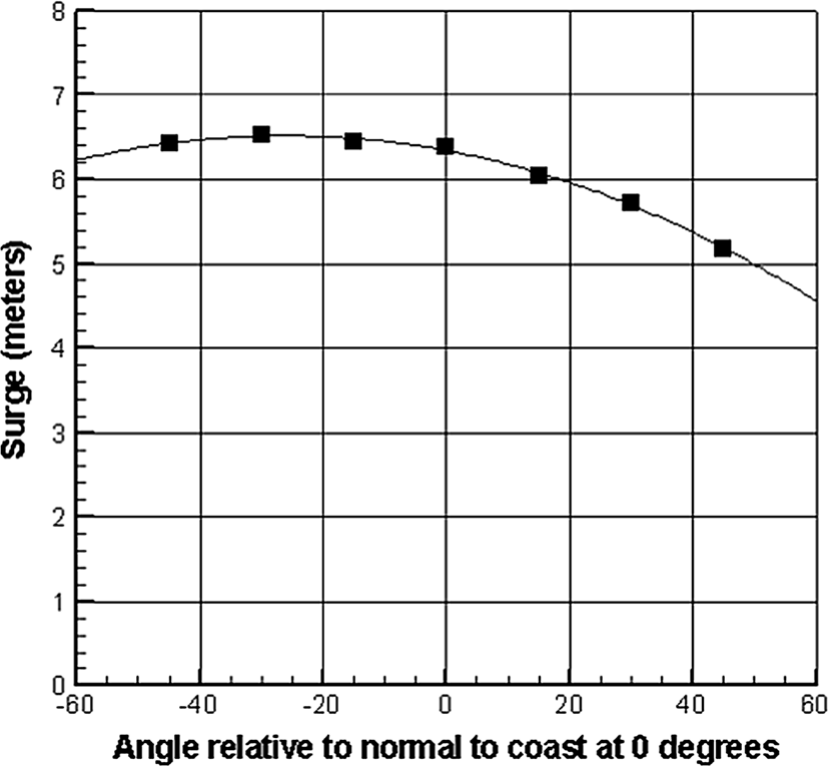
Example of a quadratic fit to surged generated by idealized simulations on a 1:10,000 offshore slope

The SRF approach, as developed in Resio et al. (2008) and Irish et al. (2009), begins with the development of consistent locally fit modeled response behavior. Next, these quantitative functions are used to make a preliminary estimate of the allowable distance between parameters needed for simulations, which is checked by subinterval testing. Figure 4 shows a typical pattern of surge variation at an idealized open coast as a function of storm approach angle. The curvature in these functions tends to be much smaller than those shown in Figs. 2 and 3, indicating that the angle spacing in the SRF can be quite broad along a uniform coast. Once all of the functions are quantified, a continuous surge response function, $\hat{\eta }(c_{p} ,R_{\hbox{max} } ,V_{f} ,\theta_{*} ,s)$, is created, where $c_{p} ,R_{\hbox{max} } ,V_{f} ,\theta_{*} ,s$ are the storm parameters, central pressure, radius to maximum winds, forward velocity of the storm, direction of storm motion and distance along the coast, respectively. This continuous response surface is then discretized into a very fine grid of surge values covering the entire JPM probability space in a dense matrix form to expedite numerical integration. In the last step, the two matrices are combined via a Heaviside function to estimate the AEP in the JPM-SRF

$${\text{AEP}}(\eta ) = \sum\limits_{i}^{{}} {\sum\limits_{j}^{{}} {\sum\limits_{k}^{{}} {\sum\limits_{l}^{{}} {\sum\limits_{m}^{{}} p } } } } (x_{i,j,k,l,m} )H(\hat{\eta }_{i,j,k,l,m} - \eta ),$$

The SRF retains information on the structure of both the response function and joint probabilities which allows them to be linked and easily interpolated. Similar to the evaluation of the accuracy in the BQ, storm parameter spacing in the SRF can be estimated objectively. Subinterval tests can be used to compare interpolations and extrapolations at a number of non-simulated parameter combinations to simulated results at both open-coast and inland points, or cross validation methods can be used.

**Fig. 5:**
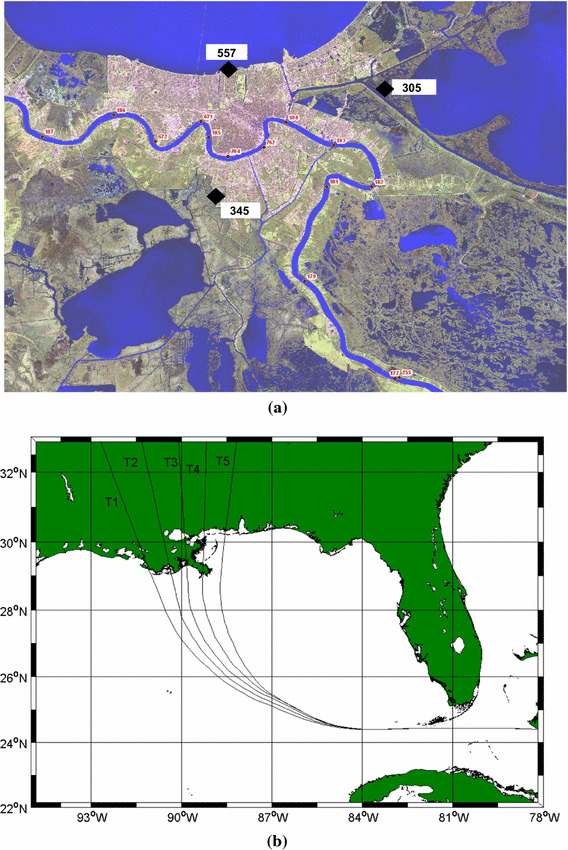
**a** Location of the test points 305, 345 and 557 and **b** the tracks use in ADCIRC surge simulations used in the New Orleans area

**Fig. 6:**
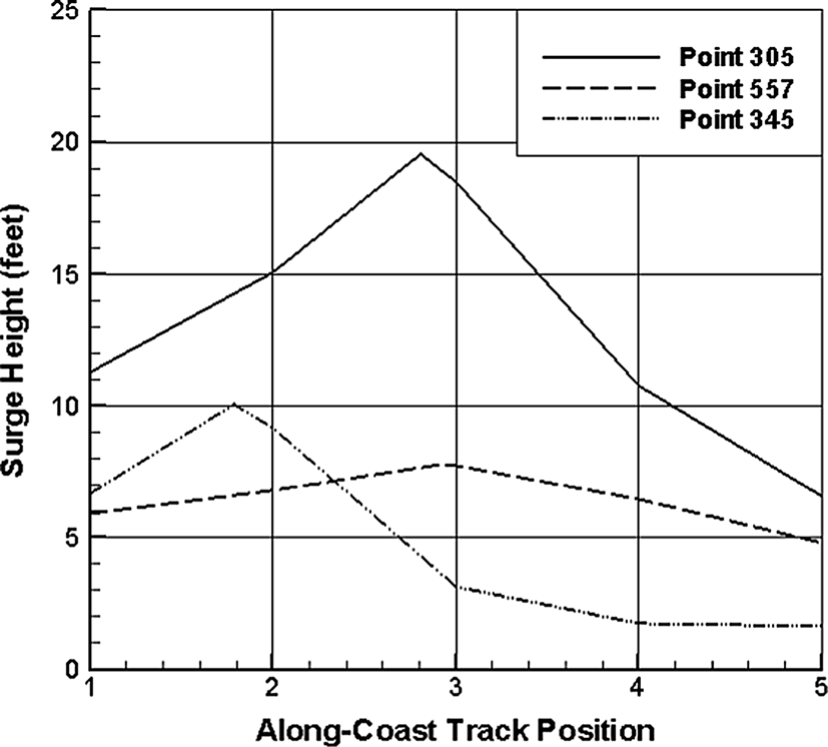
Surges produced by storms along tracks 1–5 with a maximum value estimated via Eq. 5, between adjacent points with highest surges

Resio et al. (2017) gives some examples of how these SRFs can be derived with respect to different parameters. Figure 5 (top panel) shows locations of some sample points in the New Orleans area, and Fig. 5 (bottom panel) shows the tracks used in these simulations. Figure 6 provides examples of some functional fits to surges along the coast from ADCIRC simulations in the New Orleans area, showing how these locally fit functions represent values higher than the surges at the discretized points. In applications to flood mapping, these “locally fit,” continuous functions are interpolated onto a much finer discretization than used in the hindcast storm set and can be used to provide a physical basis for extrapolation to higher values if required.

### Variations in the quantification of uncertainty

None of the methods have any inherent requirements or limitations related to the treatment of uncertainty. The 95% confidence intervals are readily calculated for fitted parametric CDFs, such as the GEV and GPD, and are commonly used with the HSM. They are developed using resampling methods in the EST. However, these only account for part of the uncertainty (the inherent sampling error in the observed data) and can often exhibit non-physical behavior, such as divergence above some threshold value. Recent applications of the BQ and SRF JPM-OS method to FEMA studies have done relatively rigorous treatments of epistemic uncertainty. In these studies, various contributions to epistemic uncertainties, such as the hydrodynamic model’s accuracy and the difference between idealized and historical wind fields, are quantified as standard deviations of a Gaussian distributed uncertainty term. A single surge elevation (whether from direct modeling in the BQ or response functions in the SRF) is then interpreted as having potentially been a range of elevations, distributed probabilistically by the uncertainty term. This is shown mathematically in Eq. 6 in Sect. 4. In more advanced cases, uncertainty terms have been used to represent multiple additional JPM parameters, such as the secondary central pressure deficit, radii of maximum winds and Holland’s B observed in historical storms exhibiting a double-exponential radial pressure profile, in order to avoid adding too many dimensions to the JPM space. As noted by Lin and Emanuel (2015), methods like the SDTM can better assess the aleatory uncertainty by extending the sampled time interval, particularly in nonstationary storm climates. To some extent, the SDTM can also allow an evaluation of the epistemic component of uncertainty by determining the range of responses yielded by using a range of different models; however, such an evaluation is not unique to the SDTM. The inclusion of uncertainty in a formalized fashion is a relatively new step in probabilistic surge hazard analyses and remains a fertile avenue for further research.

## Examples of natural structure directly affecting surge hazard estimation

### Omnidirectional sampling and storm intensity

When an omnidirectional sampling method is used as the basis for deriving a statistical distribution of a scalar quantity such as central pressure, it is inherently assumed that storm heading and intensity are independent and the probability of a combination of intensity and heading can be written as

3 $$p(\Delta P,\theta ) = p(\Delta P)p(\theta )$$

where Δ*P* is the pressure differential and is the storm heading. Physically, it is expected that overland passage, proximity to land, sea surface temperature (SST) and atmospheric shear will all affect storm intensities above some threshold. A good example of a location that restricts overwater approach angles can be found in the New York Bight. Figure 7 from Resio and Irish (2015) shows there is a strong relationship between heading and storm intensity in the historical sample of storms in this area.

**Fig. 7:**
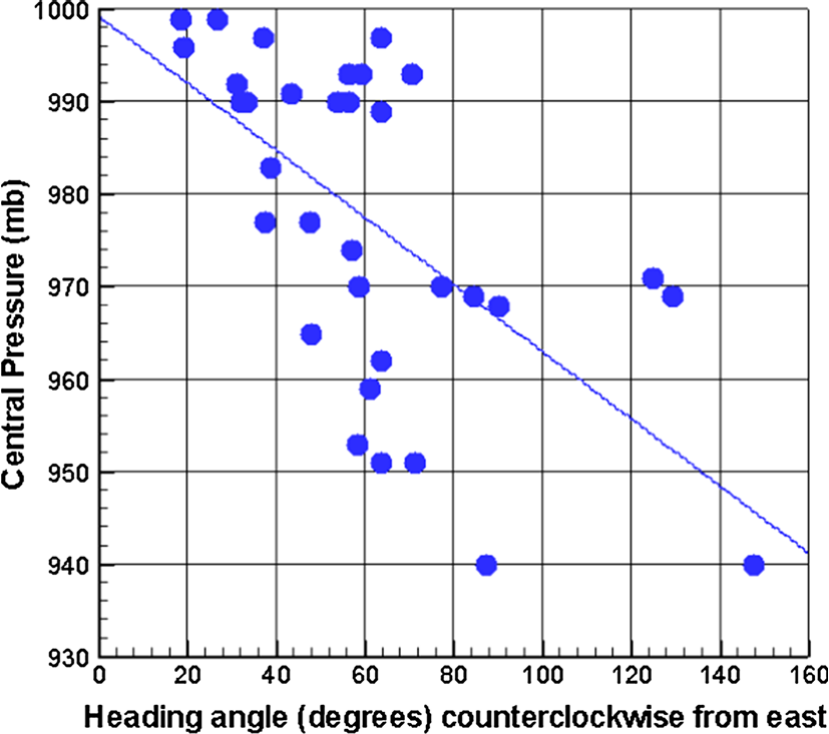
Variation of lowest central pressure in an area defined in a region bounded by latitudes from 36.5° to 41° North and longitudes between 78 − (0.222 × (Latitude − 36.5) and 73 − (0.222 × (Latitude − 36.5) West based on the latest available HURDAT reanalysis (Landsea and Franklin 2013) using all data 1930 through 2012. This defines the pressure characteristics in a region approximately the same width along the US East Coast from off the southern part of the Chesapeake Bay to the eastern part of Long Island plotted as a function of storm heading at the time of lowest pressure. The correlation is significant at the 0.01 level of significance. The angles here are heading directions measured counterclockwise from east

This type of correlation increases the likelihood of intense storms making landfall in the New York Bight, which then affects surge probabilities. In Table 1, we obtain a best-fit Poisson–Gumbel distribution using storms selected from Fig. 7, stratified to include only storms with central pressures less than or equal to 985 mb. In column B, all storms are included with intensity and heading assumed independent. If we include the effects of correlation between heading and intensity, we obtain the results shown in column C.

**Table 1:** Comparison of results from analyses of estimated storm central pressures for different data assumptions: column B—estimated central pressure neglecting heading–intensity correlation; column C—estimated central pressure including heading–intensity correlation

A: Return period	B: Central pressure neglecting correlation	C: Central pressure including correlation
20	951	965
50	940	940
100	933	922
200	926	912

It can be seen here that the two different approaches lead to significantly different results at larger return periods. The basic scaling for landfalling hurricane surges relative to central pressures developed in Resio and Westerink (2008) and Irish and Resio (2010) shows that the magnitude of a surge at an open-coast location depends linearly on the pressure differential of the storm (peripheral pressure minus the central pressure), where surge magnitude is much less sensitive to storm heading (Irish et al. 2008). Assuming the peripheral pressure is 1016 mb and that surges in this area follow this same dependency, even though some or most may be transitioning to an extratropical form, we see that surges produced by storms in column C would be about 13% higher at the 100-year return period and 16% higher at the 200-year return period than the results obtained using an omnidirectional sampling method.

### Storm intensity and storm rate

A Poisson process is assumed to represent random occurrences of events drawn from a homogeneous population. In a global sense, hurricane activity as measured by the total number of tropical cyclones around the world is relatively constant (Vecchi and Knutson 2008; Landsea et al. 2010; Vecchi and Knutson 2011; Villarini et al. 2010); yet it is well known that multi-year and longer cycles significantly affect local hurricane rates (Trenberth and Shea 2005; Chylek and Lesins 2008). This localized variability suggests storm rates are likely not constant through time in a given area. If the probability distribution of the pressure differential *F*(Δ*P*) is independent of storm rate *λ*, then the overall Poisson rate would still be applicable to the surge hazard; however, if *F*(Δ*P*) varies as a function of *λ*, the results can be quite different for extrapolated probabilities. Suveges and Davison (2010) and Fukutome et al. (2014) offer examples on how clustering can significantly affect the choice of threshold value in POT studies, their extrapolated values and their asymptotic limits.

If we examine landfalling hurricanes along all US Gulf of Mexico coastlines from 1900 through 2007, the assumption that storm occurrences per year follow a Poisson distribution is common to many compound distributions, such as the Poisson-GEV or Poisson-POT. If the population being sampled is truly homogeneous, years with few storms should follow the same distribution of intensities as years with many storms. In this case, the proportion above a fixed threshold should be constant independent of how the observations are partitioned. A simple test of this can be made by partitioning the total sample into years with a small positive number of storms and years with a higher number of storms. To maintain close to the same number of samples in each category, we chose years with one storm as one category and years with more than one storm as the two categories. Choosing a threshold of 960 mb for central pressure, we find that 28% of the storms exceed this threshold (are more intense) in years with only one storm, while 56% of the storms exceed this threshold in years with more than one storm. A simple 2 × 2 contingency table for these data is shown in Table 2. The *p* value for this table is 0.0177, which supports the argument that in years with multiple storms, the storms are stronger than in years with only one storm, or, in terms of physical argument, years with conditions favorable for hurricanes in an area are likely to have both more storms and stronger storms than years in which conditions are not favorable.

**Table 2:** 2 × 2 contingency table of storms making landfall along US Gulf of Mexico coastlines

	Years with only 1 storm	Years with more than 1 storm
Number of storms *c*_*p*_ > 960 mb	31	14
Number of storms *c*_*p*_ ≤ 960 mb	14	18

Problems with mixed populations and clustering can be found in GEV methods and have shown that both approaches have to be used with care in such situations (Ashkar and Tatsambon 2007; Dalelane and Deutscländer 2013) and as noted previously are recognized to create problems with GPD methods. Often these populations have recognizable physical bases that can help understand the differences between different populations, for example surges from extratropical storms versus surges from tropical storms and storms making local landfall versus those which make landfall some distance from a site. In cases where such an inhomogeneity is suspected and sample numbers are allowed, a more general form for mixed population should be investigated as a substitute for the single homogeneous form:

4 $$\frac{1}{{{\text{AEP}}(x)}} = T(x) = \prod\limits_{i = 1}^{n} {\frac{1}{{\lambda_{i} [1 - F_{i} (x)]}}} ,\quad {\text{where}}\quad \lambda^{ - 1} = \sum\limits_{i = 1}^{n} {\lambda_{i}^{ - 1} }$$

where *λ* is the Poisson frequency for the total of events from all populations.

## Natural structure’s effects on surge hazard estimation uncertainty

### Artificial discontinuities in the treatment of epistemic uncertainty

**Fig. 8:**
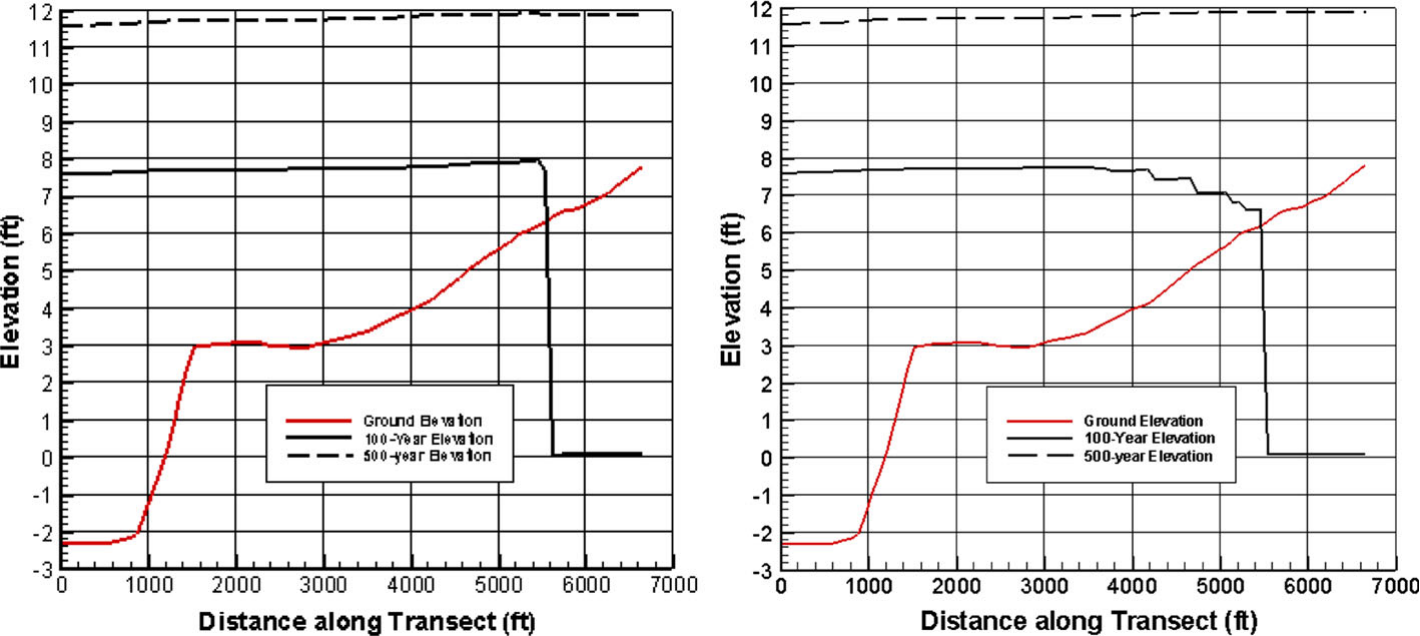
Variation in the 100-year (*right panel*) estimated still water level along a transect, with epistemic uncertainty neglected (shown as a *solid black line* in the *left hand panel*). Variation in the 100-year still water level along a transect after the inclusion of epistemic uncertainty (shown as a *solid black line* in the *right-hand panel*). *Dashed lines* provide similar information for the 500-year surge elevations

During the adaptation of the JPM-OS methodology from design applications to flood insurance applications, it became evident that the uncertainty terms (*ε*) were inadvertently creating a negative bias on shallow sloping coastal plains. An example of this is shown in Fig. 8. Determination of AEP water elevations requires integration of a water elevation probability function $p(\eta )$ and an uncertainty function $p(\varepsilon )$ as:

5 $$E\left[ {{\text{AEP}}\left( {\eta_{ * } } \right)} \right] = \int\limits_{{\eta_{ * } }}^{\infty } {\int\limits_{ - \infty }^{\infty } {p\left( {\eta |\hat{\eta } + \varepsilon } \right)p\left( \varepsilon \right)H\left[ {\hat{\eta } - \eta_{ * } + \varepsilon } \right]{\text{d}}\varepsilon {\text{d}}\hat{\eta }} }$$

**Fig. 9:**
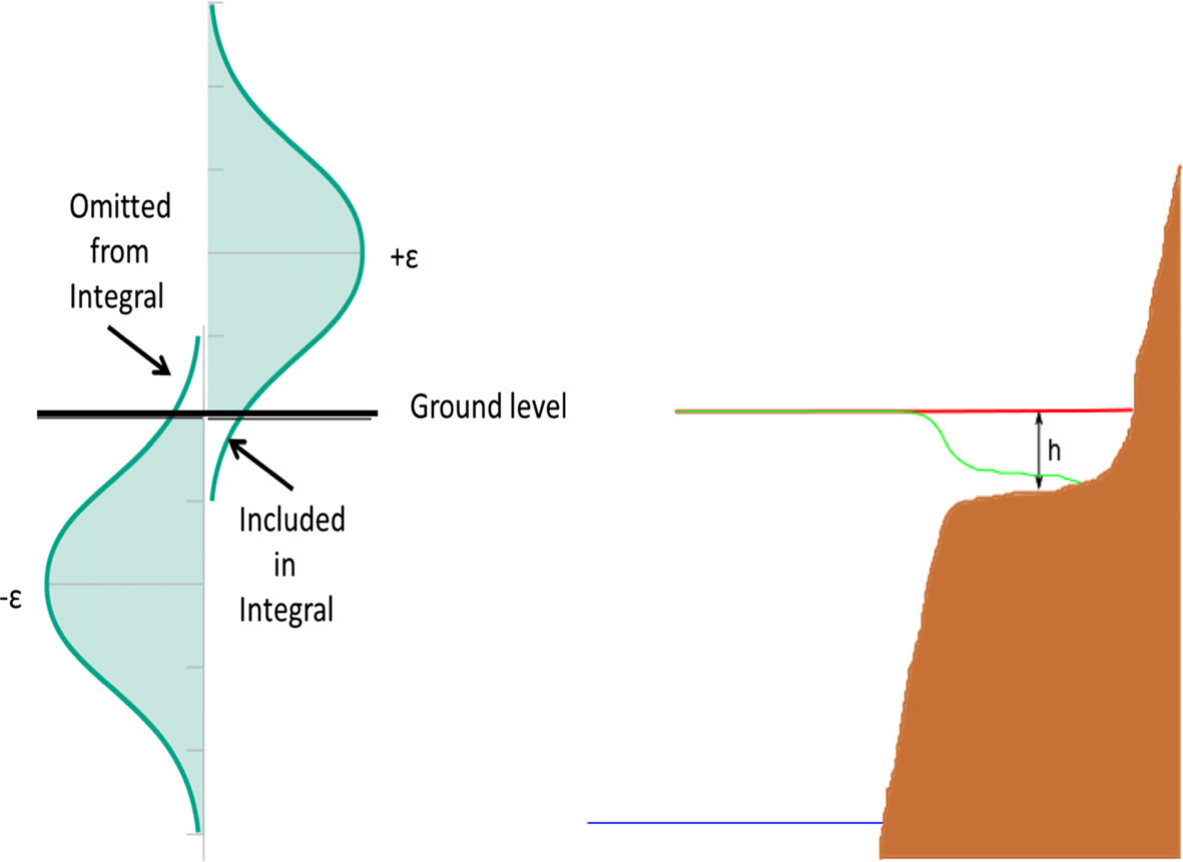
Illustration of the regions included and excluded from the total integral by the discontinuity in the addition of the epistemic uncertainty at a point. The *right-hand panel* shows a schematic of how probabilistic flood levels may be distorted. The *red line* is the “true” iso-probability contour flood level; the *green line* is the value that would be calculated when positive uncertainty contributions from surges which would inundate a point within the bounds of the uncertainty integration are neglected, while the effect of negative uncertainty contributions is retained; the *blue line* is the sea level; *h* is the flooding depth

The problem of systematic under-prediction shown in Fig. 8 is not inherent to the mathematics in Eq. 6; instead it is caused by a discontinuity (at the ground elevation) in the inclusion of epistemic effects in applications of this equation. In recent applications, epistemic uncertainty has been added to modeled storms which inundate a given point ($\hat{\eta }$ is greater than ground elevation) but not to storms that could have flooded the site when epistemic uncertainty is added ($\hat{\eta }$ is less than ground elevation, but $\hat{\eta } + \varepsilon$ is greater than ground elevation). Due to the nature of the uncertainty function, the probability of $p\left( \eta \right)$ at a water elevation below $\eta_{ * }$ can still affect $E\left[ {{\text{AEP}}\left( {\eta_{ * } } \right)} \right]$. Therefore, $p\left( \eta \right)$ must be defined over a sufficiently broad range such that any relevant part of the probability space is not lost. Figure 9a provides a simple geometric interpretation of this effect, showing that the lack of inclusion of these storms results in underestimated (low biased) statistical surge estimates. This negative bias in the statistical surge is illustrated in Fig. 9b, based on data from model simulations on a very shallow slope.

To avoid the problem of underestimation related to the omission of a portion of the epistemic uncertainty integral, $p(\eta )$ must be estimated for modeled storms that almost flood a point as well as those that do flood the point. Two methods to address this issue have been investigated. The first is based on estimation of the probabilities of storms where $\hat{\eta }$ is near, but less than, ground elevation (hereafter “under-surface”). The second is based on spatially transposing surges for storms whose simulated surge inundated neighboring points. In preliminary trials, it appears that the latter of these approaches provides a more stable approximation to the under-surface probabilities, but these two methods are still being investigated.

If we examine the mathematics governing the net gain or loss of probability at a given point where this discontinuity is not distorting the solutions, we can write the equation for the exchange of probabilities as

6 $$\begin{aligned} \delta p_{*} & = \int\limits_{{\eta_{*} - \delta \eta }}^{{\eta_{*} + \delta \eta }} {p(\eta )e^{{ - \frac{1}{2}(\eta - \eta_{*} )^{2} }} } d\eta \\ & \approx \sum\limits_{i = 1}^{n} {\varLambda (\delta \eta_{i} )\{ [p(\eta_{*} - \delta \eta_{i} ) - p(\eta_{*} )] + [p(} \eta_{*} + \delta \eta_{i} ) - p(\eta_{*} )]\} \delta \eta \\ & = \sum\limits_{i = 1}^{n} {\varLambda (\delta \eta_{i} )} [p(\eta_{*} - \delta \eta_{i} ) + p(\eta_{*} + \delta \eta_{i} ) - 2p(\eta_{*} )]\delta \eta \\ \end{aligned}$$

where $\Delta p(\eta_{*} )$ is the change in the surge pdf at the point of interest, $\varLambda_{i} (\Delta \eta_{i} )$ is the pdf of a Gaussian distribution with 0 mean and standard deviation *σ* at deviation from the mean of $\delta \eta_{i}$, $\delta \eta$ is the width of the incremental summation, and $\delta \eta_{i}$ is the location of the ith incremental element to either side of $\eta_{*}$.

As shown in Eq. 7, the form of the change in probability at $\eta_{*}$ has the same form as a diffusion equation. The probability will remain unchanged for the special case of a constant gradient in the probability density, which includes the specific no gradient in the probability densities.

If we assume that the general form of the pdf in a local area will be relatively constant, it is expected that the characteristic gradient in nearby points to a point of interest and elevation, *z*, would have approximately the same characteristic pdf above *z* and would continue the pdf to lower levels. In this case, rather than “borrowing” surges from specific storms at a neighboring point or borrowing probabilities for surges lower than *z* from nearby points and then using this information to calculate the incremental change in probability ($\Delta p(\eta )$), we could assume that $\Delta p(\eta )$ can be borrowed directly. Since these pdfs are typically of exponentially decreasing form, this operation is expected to produce a small gain in probabilities above the ground level at all points to which this is applied. Further testing of this method compared to alternative borrowing schemes is needed to clarify this issue.

### Estimation of aleatory uncertainty in the JPM considering two primary natural scales inherent in tropical cyclone surge probabilities

In a situation with two different scales of variability affecting major storms, it is essential to understand how these scales relate to each other in assessing sampling uncertainty. Irish et al. (2011) showed there was a significant reduction in uncertainty using a JPM method over using historical surge events. Here, we will investigate the role of different natural scales in creating this gain in information. Let us first assume storm speed and angle are constant to simplify this analysis. If an idealized, mathematical function representing the surge had a constant value over a fixed distance *δx* along a coast and the probability of this along-coast constant was governed by some extremal distribution, the annual encounter probability could be written as

7 $$P(\eta ) = \int\limits_{0}^{L} {\int\limits_{0}^{\infty } {p\left( {\eta |\left| {x - x_{s} } \right| \le \frac{\delta x}{2}} \right)p} } \left( {\left| {x - x_{s} } \right| \le \frac{\delta x}{2}} \right){\text{d}}\eta {\text{d}}x$$

where *η* is the surge height, $x_{s}$ is the location of the center of the region of uniform surge height in a storm, $p\left( {\eta |\left| {x - x_{s} } \right| \le \frac{\delta x}{2}} \right)$ is the probability of a surge level given a point is affected by an event, and $p\left( {\left| {x - x_{s} } \right| \le \frac{\delta x}{2}} \right)$ is the annual probability that a particular point *x* falls in a segment of coast affected by the event. Furthermore, if we assume that *L* is chosen such that the storm frequency per unit distance is constant over this length, then Eq. 8 can be rewritten as

8 $$P(\eta ) = \int\limits_{0}^{\infty } {p(\eta )\lambda } {\text{d}}\eta$$

where *λ* is a constant representing the expected number of storms per year per unit along-coast distance.

In this case, the gain in information when sampling over the distance *L* can be shown to be increased over the information acquired by sampling at a single point (i.e., the use of historical storms at a site only) by the factor $\frac{L}{\delta x}$. In this case if the along-coast length of major storm surge effects was 80 km and the length of homogeneity was 400 km, the gain in would be a factor of 5; hence, the equivalent number of years of the record would be 5 times larger than that of a historical storm surge approach. In the more general case, still holding approach angle and forward speed of the storm constant but with spatially variable surges along the coast from each storm, we have

9 $$P(\eta ) = \int\limits_{{ - \hat{x}}}^{{\hat{x}}} {\int\limits_{0}^{\infty } {p(\eta )\phi (x - x_{0} )\lambda } {\text{d}}\eta } {\text{d}}x$$

where $\phi (x - x_{0} )$ is a dimensionless SRF defining the ratio of the surge at a point of interest to the distance from landfall to that point, which has been found to be related to the size of the storm and the coastal geometry, and $\hat{x}$ is a scaled dimensionless function of the alongshore distance and the storm size (Irish et al. 2008; Irish et al. 2009). As shown by Irish and Resio (2010) (Fig. 1), this can be seen to be a functional relationship of the form

10 $$\phi (x - x_{0} ) = \phi^{{\prime }} \left( {\frac{{x - x_{0} + x^{{\prime }} }}{{R_{\hbox{max} } }}} \right) = \phi^{{\prime }} (\hat{x})$$

where $\phi^{{\prime }}$ is an SRF of two parameters, a spatial displacement factor, *x′*, and a storm size scaling parameter, $R_{\hbox{max} }$. If we now introduce variable storm approach angles and storm forward speeds, we see each of these will have a similar functional structure to Eq. 11 as shown in Irish and Resio (2010). All of these functions can be combined into a multivariate integration that provides an expected along-coast distance covered by an *n*-year event. Alternatively, in areas where secondary parameters like storm heading or forward speed are important to the response, but are not well behaved functionally, multiple independent response functions can be constructed. In such a case, the only requirement is that integration across the discrete set of response functions still reasonably approximates the (unknown) true integral, which should be achievable with a few different angles and speeds.

Limiting the treatment of functions to intensity and storm size, we see fundamental differences between the two basic scales of variability: an along-coast surge response scale that depends on storm size and a scale that depends on the size of the total along-coast sample length. The along-coast scale of variability in climatological storm characteristics is typically 100 s of km, while the along-coast scale of variability related to the surge response is on the order of 10 s of km. This relative difference in scales essentially includes more storms within the area sampled and, similar to the simple case introduced here, increases the number of samples by the ratio of these two scales. Since most distributions of uncertainty around an estimated value have a basis in the central limit theorem, most of the theoretical forms depend on some measure of the sample dispersion and the square root of the number of samples. In this context, the ratio of the spatial scales increases the number of samples and should result in a decrease in the uncertainty, approximated by

$$\sqrt {\frac{{\left\langle {\delta x_{\text{L}} } \right\rangle }}{{\left\langle {\delta x_{\text{s}} } \right\rangle }}}$$

where the brackets denote an average value of the width of the two scales, with “L” denoting the larger, climatological variation scale and “s” denoting the smaller “surge response” scale along the coast. Predictions based on this concept are consistent with the Monte Carlo simulation results presented in Irish et al. (2011).

This ratio of scales potentially depends on the annual exceedance probability (AEP), so the actual averaging for this application must be done numerically. If we assume the scale for the climatological variability is obtained by methods similar to Chouinard et al. (1997), we can use that as a reasonable estimate of $\left\langle {\delta x_{\text{L}} } \right\rangle$, but the estimate of $\left\langle {\delta x_{\text{s}} } \right\rangle$ must be obtained from an integral of the form

11 $$\begin{aligned} \left\langle {\delta x_{\text{s}} ({\text{AEP}})} \right\rangle & = \int\limits_{{}}^{{}} {\int\limits_{{}}^{{}} {\int\limits_{{}}^{{}} {\int\limits_{{}}^{{}} {\int\limits_{{}}^{{}} {p(\delta x_{\text{s}} )p(c_{p} ,R_{\hbox{max} } ,v_{f} ,\theta )} } } } } \\ & \quad H[\varLambda (c_{p} ,R_{\hbox{max} } ,v_{f} ,\theta ,\delta x_{\text{s}} ) - \eta (AEP)]{\text{d}}c_{p} {\text{d}}R_{\hbox{max} } {\text{d}}v_{f} {\text{d}}\theta {\text{d}}\delta x_{\text{s}} \\ \end{aligned}$$

where the integration over $\delta x_{\text{s}}$ can be seen to yield a width of the region contributing to that exceedance probability, and Λ is a surge response function. This equation states that the mean value of $\delta x_{\text{s}}$ at a given AEP results from the integration of a probability distribution and another of the tropical cyclone parameter space. The Heaviside function serves to ensure that storm parameter combinations that yield surges below $\eta ({\text{AEP}})$ do not contribute.

### The effect of natural scaling on epistemic uncertainties

In tropical storm hazard estimation today, it is common to assume that the magnitude of the epistemic uncertainty is independent of the magnitude of storm surge (Resio et al. 2008; Irish et al. 2009; Toro et al. 2010; Niedoroda et al. 2010), with the exception of Holland’s B, which was accounted for as an uncertainty term that scaled with the storm surge in several of these recent studies. In the initial formulation by Resio et al. (2008), the Holland B was grouped into a number of factors which were intended to incorporate the deviations between parametric and “real-world” winds related to phenomena such as wind-field asymmetry, variations in intensity during landfall, wind-field perturbations due to spiral bands and other details neglected in the parameterization; this term should not be interpreted as only the deviations caused by neglect of the Holland B term.

The inclusion of this form for epistemic uncertainty was initially prompted by the detailed results of hindcast surge comparisons from Hurricanes Katrina and Rita in 2005; however, that work was focused on the development of a probabilistic tool for levee design in areas that had been devastated by these storms. In this context, the assumption was made that the magnitude of epistemic uncertainty was independent of surge magnitude. Whereas an acceptance of this assumption might be very justifiable in a design application (where the focus is on flooding at a specific, usually high elevation), it might not be justifiable in cases where a broad range of flood magnitudes are of interest, such as actuarial applications. Surge magnitudes are expected to be related to the size and depth of the surge generation area and wind forcing in these areas (Resio and Westerink 2008). For example, in a small lake the same wind and pressure forcing generate surges which would scale with the size of the area over which the forcing acts (Irish and Resio 2010). If uncertainty is not allowed to scale with the dimensions of the phenomena under investigation, natural scaling factors, inherent to the equations governing surge equations, are being neglected.

Due partly to data quality limitations, the validity of this assumption (i.e., assuming that the standard deviation of uncertainty is essentially constant) has not been assessed robustly. Uncertainty quantifications have generally been performed by comparing modeled surge estimates under varying circumstances. In the case of hydrodynamic and meteorological model errors, a comparison between modeled and measured water levels is used. Most hurricane surge observational datasets (including the comparisons done for Rita and Katrina) come from post-storm surveyed HWMs, for which the measurement error is typically larger than the surge model error. It should be noted, however, that this can be, and has been, accounted for in bulk statistics; the 0.23 m standard deviation in model uncertainty stated by Niedoroda et al. (2010) comes from subtracting the variance seen in tightly clustered HWMs (i.e., measurement error) out from the overall variance between modeled and measured surges.

### Potential problems with stochastic-deterministic models used for very long simulation periods to reduce aleatory uncertainty for low-probability storm surges

#### Potential problems with applications of models with bias

As shown by Resio et al. (2013), the estimation of very low probabilities is strongly affected by uncertainty in estimated values. The overall expectation of an exceedance was written in that paper in terms of a delta function, but is rewritten here in terms of a Heaviside function

12 $${\text{AEP}}(\eta ) = \int\limits_{0}^{\infty } {\int\limits_{ - \infty }^{\infty } {p(\eta_{*} + \varepsilon )p(\eta_{*} )p(\varepsilon } |\eta_{*} )H\,[} \eta_{*} + \varepsilon - \eta ]{\text{d}}\varepsilon {\text{d}}\eta_{*}$$

where *ε* represents a continuous uncertainty variation around the modeled value (assumed to follow a Gaussian distribution with zero mean and a specified standard deviation), $\eta_{*}$ is the dummy surge integration parameter, and $\eta$ is the surge value for which the AEP is estimated. As noted previously, here we consider the epistemic component of uncertainty to include parameterization of storm wind fields, omission of (or approximation of) astronomical tides, storm track variations not explicitly considered and hydrodynamic modeling errors, among other factors. Results from that study show the potentially high impact of aleatory uncertainty on the estimation of storm central pressures as the return period information from estimates becomes large relative to the period of record used to define storm characteristics.

Lin et al. (2010) demonstrated an approach intended to reduce the impact of the aleatory uncertainty by using synthetic storms (Emanuel et al. 2006). The approach provides a mechanism for increasing the number of synthetic storms and years sampled and an ability to include climate variability into these estimates. However, due to the very large number of storms generated for a single application, this model used in this approach is limited to low-resolution, small-domain applications, which can create significant bias when compared with the high-resolution surge model results (Lin et al. 2010). In this case, Eq. 13 becomes

13 $${\text{AEP}}(\eta ) = \int\limits_{0}^{\infty } {\int\limits_{ - \infty }^{\infty } {p(\eta_{*} + \varepsilon )p(\eta_{*} )p(\varepsilon } |\eta_{*} )H\,[} \eta_{*} + \beta (\eta_{*} ) + \varepsilon - \eta ]{\text{d}}\varepsilon {\text{d}}\eta_{*}$$

where $\beta$ is the bias term introduced by the model. This term can substantially alter the surge probabilities in both forecast and hindcast surges (Resio et al. 2017).

#### Potential problems with transitioning extratropical storms

It will be also important to validate the Emanuel et al. (2006) synthetic hurricane wind model against the Holland (1980, 2010) model or planetary boundary layer models (Thompson and Cardone 1996; Vickery and Twisdale 1995), since the latter two wind models have been validated in terms of their capability to produce unbiased estimates of surges in historical storms. This complicates the need to quantify the epistemic uncertainty (the state-of-the-art modeling biases and random errors) related to interrelationships among all the forcing factors neglected in an idealized model that simulates synthetic storms, such as hurricane–land interaction which can be critical at landfall and complex multivariate correlation structures.

A factor of particular importance is the neglect of baroclinic energy sources in the overall energy balance, as noted in the review by Resio and Irish (2015). Hurricane Sandy was transitioning into an extratropical system when it approached landfall, drawing about 50% of its energy from baroclinic sources. The 1938 storm that devastated much of Long Island was also transitioning to an extratropical form, as was Hurricane Irene in 2011. Jones et al. (2003) suggests more than 50% of the tropical cyclones north of 35° latitude have begun extratropical transition before landfall. For this reason, it seems the use of synthetic storms that neglect this energy source in an area north of 35° could lead to serious underestimation of expected hurricane intensities. An interesting side note on the tracks of the 1938 storm and Hurricane Sandy is both of these storms underwent retrograde motion, with their tracks curving westward into the Great Lakes region, as they were absorbed into larger-scale extratropical circulation patterns; thus, these storms were unique when compared with the majority of tropical systems passing by the New York coast that recurve out to sea. The neglect of baroclinic energy sources raises serious concerns with the application of the SDTM to US coastlines north of 35°N, since the limiting intensity for the maximum intensity is missing in the primary approximations used in such models (Emanuel 1988; Holland 1997; Emanuel et al. 2004).

Discussions in this section raise an important question concerning whether or not information added from a synthetic storm population outweighs the uncertainty added. A historical dataset is subject to uncertainty regarding the sufficiency and representativeness of the dataset (aleatory uncertainty), but a synthetic storm dataset can be affected both by the model skill (epistemic uncertainty) and by the representativeness of the data used to develop empirical coefficients within the model (aleatory uncertainty). Inevitably, there will be situations under which the method either increases or decreases modeled uncertainty. Given these concerns, a robust assessment of the technique in this respect is needed.

## Conclusions

We began this paper with a brief review of the evolution of methods used for characterizing tropical cyclone surge hazards over the last fifty years. In spite of this evolution, the understanding of the potential role of natural structure on hazards has remained a somewhat unexplored field. Instead, recent progress in advancing understanding of statistical variability has focused on investigations of various types of extremal and other distributions and methods of optimal fitting methods for these distributions. The influence of inherent spatial and temporal organization on these distributions has, for the most part, been neglected. In this context, natural organization is assumed to be implicit in the distribution parameters, assuming that the available sample is, in a general sense, reasonably treated within the framework of a single, homogeneous population. However, natural organization tends to contain multiple scales, which interact and often cannot be represented by a simple one-, two-, or three-parameter form.

Following this review, we presented an investigation focused on improving our understanding and quantifying the role of natural structure as it impacts both the estimation of extremes and the uncertainties in these extremes. We found that this approach provides valuable information on potential extremes and offers a valuable complementary perspective that provides a physical basis for observed behavior in many situations. We also found that care must be taken to distinguish between statistical approaches using discrete samples (probability masses) and those using defined continuous probabilities densities (pdfs). Toward this end, we introduced examples of such differences in the two methods most often used for coastal flood mapping in the USA: Bayesian quadrature (BQ) and surge response functions (SRF). It is not clear which of these methods offers the best option to meet future needs for quantifying coastal resilience, since detailed comparisons of the two methods have not be made to date.

We also noted that natural limits on some hurricane parameters, such as maximum intensity and size, are still subject to considerable epistemic uncertainty. Such limits can affect distributions of storm intensity and size on very local scales and are likely to be dependent on many local factors, such as proximity to land, variations in sea surface temperature and variations in large-scale circulation patterns. A good example of this complexity is the space–time pattern of variability in storm parameters for tropical cyclones approaching a coast. Rappaport et al. (2010), in an analysis of all tropical cyclones making landfall on the US Gulf coast, found that during their final 12 h before landfall, hurricanes underwent different systematic patterns of evolution that depended on their initial intensity 12 h prior to landfall. In the net, category 1–2 hurricanes strengthened, while category 3–5 hurricanes weakened. Resio et al. (2008) and Levinson et al. (2010) found similar trends in their analysis of pre-landfall weakening in tropical cyclones. This behavior potentially presents problems in areas within the Gulf of Mexico that are dominated by weaker hurricanes. In such areas, it would be expected that the weaker storms exhibit little or no pre-landfall increase in central pressures when approaching land; however, caution should be exercised before such behavior is interpreted to be valid for the higher-intensity storms likely to be used for quantifying overall coastal hazards.

In this paper, we also examined examples of how the neglect of natural structure may influence estimates of coastal surge hazards generated by tropical cyclones. Our primary findings relative to these investigations include the following:

1. Omnidirectional sampling was originally developed and tested for functionality for wave generation in the Gulf of Mexico (Chouinard et al. 1997). Wave generation capacity is expected to be independent of wind direction at sites removed from the coast; however, storm surges are primarily a coastal phenomenon and depend strongly on proximity to the coast. Consequently, the near-coast behavior of storms, which include potential physical interactions influencing storm characteristics, can create correlations between storm intensity and heading that should be investigated on a local basis, particularly in areas with large-scale variations in coastline geometry.
2. Clustering of tropical cyclones in time and space, such as that driven by large-scale circulation patterns and climate variability, is expected to affect estimated surge AEPs, as will non-stationarity and coastal evolution. These are complex problems to address; but given the importance of the accuracy of such estimates, this is a valuable research area that should continue to be developed.
3. The initial method used to incorporate epistemic uncertainty into surge results was developed for design applications in which each event inundated all the sites of interest. When applied to inland sites inundated by only a portion of the simulated storms, it is essential to modify the methodology to avoid introducing artificial lowering of estimated AEPs at these sites. A method suggested here is perhaps a simple, yet sensible approach to this problem, but more research is needed to investigate the accuracies of different approaches to solving this problem.
4. In the incorporation of aleatory uncertainty into hazard/risk estimates, it is essential to quantify the increase in information provided by the JPM approach, due to the disparity in the spatial scale of variation in surge responses to an individual storm and the spatial scale of statistical variation of storm probabilities.
5. The assumption that epistemic uncertainty is scale-independent is inconsistent with the natural scaling within the equations governing surge generation.
6. Although the SDTM provides the ability to increase the number of years in a sample, it is important to quantify the epistemic uncertainty to confirm that the generated surges from tropical cyclones provide an overall gain in information for estimating the surge AEPs. Thus, a comparison of the potential reduction in the aleatory uncertainty relative to the increased epistemic uncertainty, created by either using overly simplified surge models or idealized storm evolution models that neglect many localized factors, is essential to the understanding of how this approach can best be utilized.
7. The evolution of methods to assess flood hazards has brought forth several advanced techniques whose strengths and weaknesses have not been well documented. This paper provides a qualitative assessment of the many approaches and points to several ways in which the existing methods may be able to complement each other. The potential for the SDTM and JPM-OS methods to complement each other, in particular, may be very fruitful and should be studied further.
8. It is noted in Sect. 3 that fundamental differences exist between methods based on probability masses (HSM, EST, SDTM and JPM-OS-BQ) and methods in which simulated surges represent points within a continuous multivariate space (JPM-OS-SRF). In addition to the difference in event interpretation discussed in that section, some other differences in approaches to coastal hazards that should be investigated are noted below.
  - Probability mass methods do not allow for interpolation between samples, unless the estimates are converted to points within the continuous multivariate probability field and the information is interpolated within an SRF context. In some locations with a low number of inundations, the inability to interpolate leads to very jagged AEP curves. Although these are smoothed some by the addition of the epistemic uncertainty, such smoothing may not be consistent with physically based AEP interpolations. On the other hand, interpolations are very direct in the SRF approach. This serves as both an advantage and a disadvantage, since fitting SRFs to surge could be challenging under some cases.
  - Methods which optimize the selection of storms based on a probability convergence, such as the BQ method, effectively produce a defined coupling between the multivariate probability distribution and the simulations. If any of the probability characteristics change significantly, the storm set may have to be reoptimized and the new set of storms resimulated. Methods not based on an optimized convergence are essentially uncoupled with the storm set, and the probabilities can be changed without the need to rerun a new set of simulations.
  - Conversely, any SRF approach is inherently more sensitive to the physical system, since surge results from individual simulations affect the fitted functions as a whole. This means that changes to the physical system, such as the addition of flood control structures or sea-level rise studies, may require the SRFs and the surge simulations used to create them be reevaluated. The BQ approach does not have such a strong dependency.
  - Because of inherent nonlinearities in surge response, in the SRF approach the return periods clearly depend on the size of the increments used to interpolate the event, which should be limited to a range for which the interpolations/extrapolations remain accurate. In early applications, the SRF method was exercised using no a priori information in the storm selection process. Now that it has become customary to perform this large set of low-resolution surge modeling runs to obtain such information, and it is clear that the SRF method should use it to test for convergence of the selected optimal storm set for high-resolution surge simulation to the a priori large set of low-resolution simulations. This extension to the existing SRF methodology will be investigated in part 3 of this paper.

These and several other issues have the potential to impede the progress of coastal storm surge flood hazard analyses. Examples of additional complications in these analyses include incorporation of coastal erosion during storms, improved treatment of waves in overland environments, combining inland flooding sources and a more robust representation of non-tropical storm sources. The issues addressed in this paper focus primarily on the statistical aspects of storms, because this is thought to be the area of greatest uncertainty (due largely to the data limitations). Therefore, the most pressing issue may be an assessment of how synthetic climatological datasets like those developed in the SDTM can be utilized, by combination with SRFs, for better defining the probability structure of JPM approaches. It must again be stressed, however, that proper quantification and inclusion of uncertainties in such an approach are critical to any evaluation of its utility.

It is likely that continuing economic development and population growth in coastal regions, combined with the geomorphic evolution of the coast and climate variability, will make resilience and sustainability in these areas a critical global issue. This study represents only a preliminary investigation into the effect of natural structure on tropical cyclone surges around the world. Yet, even in the limited context shown here, neglecting such natural structure appears to have a significant effect on the hazard probabilities, suggesting that this should definitely be included in future hazard assessments.

This paper is intended to be the first in a series, with the additional papers delving deeper into the effects of natural structure in two specific areas. The second paper will provide a more fundamental investigation into differences between discrete probability methods, such as the JPM-OS-BQM, and continuous probability distributions, such as the JPM-OS-SRF, as part of the analysis will examine the potential of hybrid combinations of the BQ and SRF variants to be combined into a single hybrid method. The third paper will investigate the natural scaling inherent in epistemic model errors, while assessing methods for quantifying and including uncertainty in flood hazards.
